# Gonorrhœa Caused by Leucorrhœa

**Published:** 1866-09

**Authors:** Benjamin Durham

**Affiliations:** Chicago, Ill.


### *Gonorrhœa Caused by Leucorrhœa.* By Benjamin Durham, Jr., M. D., Chicago, Ill.

(Read before the Chicago Medical Society.)

Even in the medical profession there is a wide-spread empiri cism with regard to venereal affections. The first application for professional advice made to the medical student is for some remedy which will safely, secretly and quickly remove all symptoms of a "private disease;" and through life there generally is an unrecognized tendency to consider these disorders as entirely dissimilar to derangements in other portions of the system, since their origin is generally disgraceful, and very many receive with scorn the opinion that a man may contract one of the diseases of this class, viz., gonorrhœa, without transgressing the rules of morality. On account of this early prejudice, some rarely think of this affection as an inflammation of the mucous membrane of the urethra which has proceeded to suppuration, and which often is increased by the irritation of the urine in its passage. But viewing it in this light, we cannot avoid the conclusion, that while it is generally produced by connection with a person previously having the same disease, yet it may occur among persons of virtuous habits, and on this point we must receive as conclusive the evidence on which we can rely in regard to other concerns of life. In regard to the cause of gonorrhœa, patients will generally lie, and yet sometimes will tell the simple truth, which is irresistible, because supported by the circumstantial evidence. Recently two cases have come under my observation, where I am willing to place implicit trust in the parties.

A lady consulted me in regard to the possibility of her causing an urethral discharge in her husband. She was well advanced in lactation, had not menstruated since the birth of her child, was in good health, and enjoyed quite moderate sexual intercourse as both husband and wife were watching for the return of her menses. Two days after marital connexion her husband complained to her of a slight itching heat of the glans penis, and four days after of a discharge. He consulted a physician, who told him he had the gonorrhœa, and prescribed some remedies which readily cured him. The wife then noticed a slight leucorrhœal discharge which she had not thought worthy of attention; but as her husband wished her to use the medicine prepared for him, together with vaginal injections, every trace of disease passed away, though she was unwilling to believe that such a slight discharge could cause a gonorrhœa, when years before, a severe uterine leucorrhœa, accompanying an ulceration of the os uteri, had not affected her husband. Still she had no cause to doubt her husband's purity, especially as he had made no attempt at concealment.

Again, a gentleman of good character complained to me of an irritation of the glans penis, which I supposed was a casual irritation, and so without examination I directed him to syringe out the urethra with cold water, but he returned in two days with a free gonorrhœal discharge. Nine days previous to his first calling he had connection with his wife, and that was the only known cause for this disease. She supposed herself perfectly well, was pregnant, and had not suspected any trouble with herself, though she too had previously been treated for ulceration of the os uteri, and at that time the leucorrhœa had not produced any urethral discharge from her husband.

Before gentlemen sneer at the truth of these statements, let them remember that no means of investigation as yet in our power can discriminate between the suppurative discharges from an inflamed urethra and those from similar mucous membranes. The gonorrhœal discharge consists in an alteration of the urethral mucus, (sometimes combined with the secretion of contiguous glands,) which has been called by Ricord "muco-pus," and not only may be originated by lawful connection but even without sexual intercourse. Hunter knew of the urethra repeatedly sympathizing with the cutting of a tooth with all the gonorrhœal symptoms, and likewise in cases of gout and rheumatism. Ricord holds similar views. Bumstead quotes Ricord, Harrison and Latour as teaching the production of gonorrhœa by tubercular deposit, scrofulous diathesis, free indulgence in fermented liquors, terebinthinate medicines, eating asparagus, and prolonged sexual excitement as in the case of a physician who, for nine hours, vainly attempted to overcome the virtue of a woman who resisted all his approaches. But especially does Bumstead bring up proofs of the subject before us, on which Ricord says, "Gonorrhœa often arises from intercourse with women who themselves have not the disease. Any one who studies gonorrhœa without preconceived notions is forced to admit that it often originates from the same causes that give rise to inflammation of other mucous membranes." Diday says, "A man should never forget that gonorrhœa may be contracted from *any* woman." Fournier thinks "gonorrhœa is less frequently contracted from contagion than from excessive coitus." Bumstead quotes Mr. Thompson, Mr. Skey, and especially Dr. B. F. Barker, who says he has noticed in *numerous* instances in married life urethritis produced by an acid and acrid discharge from the uterus. Every one who will give this subject much thought will find instances where gonorrhœa originates from coitus at or near the menstrual period, or where the woman is suffering from leucorrhœa. But here the inquiry may be raised—Why then does not gonorrhœa result oftener from marital intercourse? To this we may answer, that many men are afraid to trust their physicians with a recital of their cases lest a suspicion of impurity may be aroused; secondly, lawful connection is generally without excessive excitement and not long continued; thirdly, married ladies are generally particular at their toilet to remove leucorrhœal discharge by the use of the syringe; and lastly, the urethra, as well as other organs, becomes habituated to the presence of irritants, and thus escapes infection.

